# *Mycobacterium abscessus* keratitis after LASIK surgery

**DOI:** 10.5935/0004-2749.2021-0235

**Published:** 2022-09-06

**Authors:** Rengin Aslıhan Kurt, Deniz Arik, Nilgun Yildirim, Afsun Sahin

**Affiliations:** 1 Department of Ophthalmology, Istanbul Hospital, Faculty of Medicine, Baskent University, Istanbul, Turkey; 2 Department of Pathology, Faculty of Medicine, Osmangazi University, Eskisehir, Turkey; 3 Department of Ophthalmology, Faculty of Medicine, Osmangazi University, Eskisehir, Turkey; 4 Department of Ophthalmology, Faculty of Medicine, Koç University, İstanbul, Turkey

**Keywords:** Cornea/microbiology, Corneal ulcer, Eye infections, bacterial, Mycobacterium abscessus, Refractive surgical procedures, Keratomileusis, laser in situ, Amikacin/therapeutic use, Clarithromycin/therapeutic use, Humans, Case reports, Córnea/microbiologia, Úlcera da córnea, Infecç ões oculares bacterianas, Mycobacterium abscessus, Procedimentos cirúrgicos refrativos, Ceratomileuse assistida por excimer laser in situ, Amicacina/uso terapêutico, Claritromicina/uso te rapêutico, Humanos, Relatos de casos

## Abstract

A 33-year-old male presented with unilateral subacute infectious keratitis 4 weeks after surgery. Corneal inflammation was resistant to standard topical antibiotic regimens. During diagnostic flap lifting and sampling, the corneal flap melted and separated. Through flap lifting, corneal scraping, microbiological diagnosis of atypical mycobacteria, and treatment with topical fortified amikacin, clarithromycin, and systemic clarithromycin, clinical improvement was achieved.

## Abstract (pt)

Paciente do sexo masculino, 33 anos, apresentou ceratite infecciosa subaguda unilateral 4 semanas após a cirurgia. A inflamação da córnea foi resistente aos regimes de antibióticos tópicos padrão. A aba da córnea foi derretida e seccionada durante o levantamento e amostragem para diagnóstico. A melhora clínica só foi alcançada após levantamento do retalho, raspagem e diagnóstico microbiológico de micobactérias atípicas e tratamento com amicacina fortificada tópica, claritromicina e claritromicina sistêmica.

## INTRODUCTION

Atypical mycobacterial infections of the cornea are rare but serious^(1,2)^. Generally, they occur a few weeks after LASIK surgery, and the common source of infection is contaminated microkeratome equipment used for flap creation^(3)^.

## CASE REPORT

A 33-year-old male presented with blurred vision and redness in his left eye 4 weeks after LASIK surgery performed elsewhere. His medical records included a preoperative visual acuity of 20/20 with a manifest refraction of −3.75 −0.50 × 180 D. During surgery, a corneal flap was created using a microkeratome, and excimer laser treatment was applied. Postoperatively, he began receiving topical moxifloxacin and dexamethasone eye drops. On postoperative day 1, the visual acuity was 20/20. Additionally, slit-lamp examination detected no abnormalities, and no other ocular and systemic diseases occurred.

**Figure 1:**
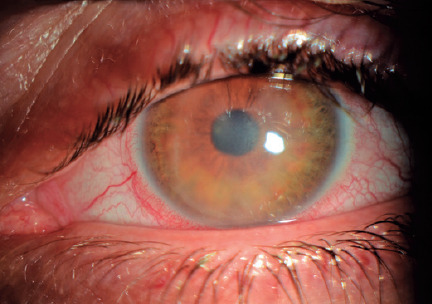
Conjunctival +3 hyperemia and difuse corneal haze at week 4 of LASIK surgery.

Upon admission to our clinic, his visual acuities were 20/20 and 20/60 in the right and left eyes, respectively. In the slit-lamp examination, his right eye had a clear cornea with regular LASIK flap borders. However, his left eye revealed conjunctival injection, intense hyperemia, and diffuse corneal haze, but no epithelial defects or corneal ulceration were noted (Figure 1). In both eyes, fundus examination and intraocular pressures showed no abnormalities. Flap lifting, corneal scraping, and interface irrigation with fortified antibiotics were subsequently recommended. Unfortunately, the patient refused. Instead, topical moxifloxacin was started, and then he decided to leave.

**Figure 2:**
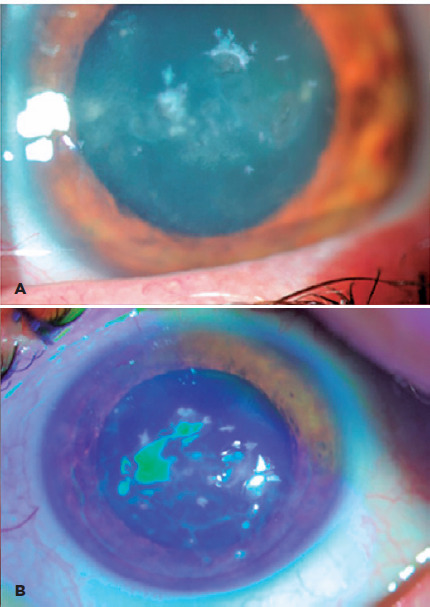
Second clinical presentation of the patient after 6 weeks. A) Multiple corneal crystalline deposits and difuse corneal haze. B) Accompanying epithelial defects.

**Figure 3:**
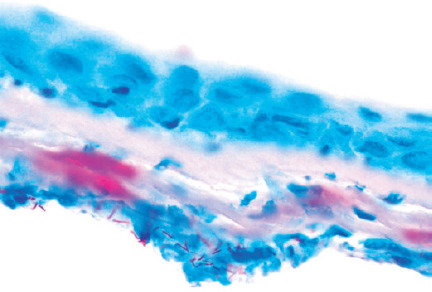
Pathological acid-fast staining of the autoamputated fap shows multiple *Bacillus* microorganisms.

Six weeks later, he complained of deteriorated vision and increased symptoms. During this time, he had been applying moxifloxacin eye drops every hour. On examination, the best-corrected visual acuities (BCVA) were 20/20 and 20/200 in the right and left eyes, respectively. Left-eye biomicroscopy showed multiple corneal crystalline infiltrates under the corneal flap (1/3 corneal thick ness) with overlying epithelial defects and increased haze in central and paracentral corneal areas (Figures 2A, B). With patient’s informed consent, we lifted the left-eye corneal flap completely, scraped the corneal stromal bed, and collected a specimen for microbiological culturing. We did not find any other corneal infection case from patients who underwent refractive surgery on the same day and place. Subsequently, the flap-stromal bed interface was irrigated with vancomycin (50 mg/mL) and amikacin (25 mg/mL). During flap lifting, the corneal flap was autoamputated, partially because of intense corneal melting. The amputated flap was then sent for pathologic evaluation. However, microbiological evaluations performed with Gram, Giemsa, and acid-fast staining did not reveal the infectious agent. Thus, he was treated empirically using fortified topical vancomycin (50 mg/mL) and amikacin (25 mg/mL) every hour and oral tetracycline (100 mg) twice daily. On day 4 of treatment, histopathologic examination detected multiple acid-resistant bacillus within the autoamputated flap specimen (Figure 3). Hence, the treatment regimen was changed to topical clarithromycin (10 mg/mL) and amikacin (25 mg/mL) every hour and oral systemic clarithromycin (500 mg) twice a day. Within 48 hours, the patient improved both symptomatically and clinically. After 2 weeks, the diagnosis was confirmed with the positive growth of *Mycobacterium abscessus* in the Lowenstein-Jensen agar.

**Figure 4:**
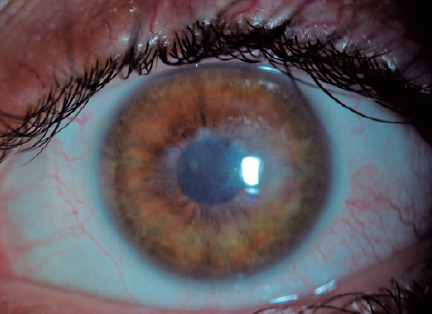
Mild corneal haze at month 8 of follow-up with the best-corrected visual acuity of 20/63.

At week 2 of treatment, only a mild corneal haze remained, without any crystalline infiltrates and epithelial defects. The treatment was tapered carefully and discontinued in 3 months. At month 8 of follow-up, the left-eye BCVA improved to 20/63, and only mild corneal haze was detectable on slit-lamp examination (Figure 4).

## DISCUSSION

Infectious keratitis after LASIK surgery is a serious complication with an estimated incidence of 1.5%, and 47% of the cases are caused by mycobacteria^(2)^.

Prophylactic use of topical fluoroquinolone drops for a couple of days adequately protects the cornea during the epithelial healing period. This routine certainly applies to surgeries performed under conditions with strict aseptic sterilization rules and use of sterile surgical equipment. The most frequently isolated subtypes of atypical *Mycobacterium* in post-LASIK keratitis are *M. chelonae* and *M. fortuitum.* However, *M. abscessus, M. mucogenicum, M. terrae, M. szulgai,* and *M. intracellulare* are rarely reported^(3,4,5)^. Symptom onset is generally at 3-10 weeks, but *M. chelonae* and *M. abscessus* may present earlier because they are rapidly growing micro-organisms^(3,6)^. Usually, serious atypical mycobacterial corneal infections are reported after improper sterilization of the surgical equipment or reuse of disposable microkeratome blades^(3,5)^.

Patients generally present with blurred vision and red and irritated eyes, which do not respond to the routinely used topical antibiotics and steroids, a few weeks after LASIK surgery^(3,6)^. The late onset and crystalline deposition in the interface may alarm the physician for mycobacterial keratitis^(7)^. To avoid treatment delays, physicians should not confuse early signs of myco-bacterial keratitis in a post-LASIK patient with diffuse lamellar keratitis, which responds well to topical corticosteroids^(3)^. Differential diagnoses must also include herpes keratitis, *Nocardia, Acanthamoeba*, and infectious crystalline keratopathy because they may cause nonsuppurative keratitis^(6)^.

As in all post-LASIK keratitis, the initial step of diagnosis and treatment includes flap lifting, corneal scraping with culture, and interface irrigation with antibiotics^(1,2,3,6)^. The standard microbiologic workup for a patient with presumed *Mycobacterium* infection includes acid-fast staining and plating on the Lowenstein-Jensen agar^(7)^. However, given that the growth of the pathogen in the Lowenstein-Jensen agar may take time (up to 8 weeks), treatment should be started with topical amikacin, clarithromycin, and fourth-generation fluoroquinolones^(3,6,7)^. Moxifloxacin and mild levofloxacin and ciprofloxacin have been reported to be significantly effective in reducing the number of *M. abscessus* in vivo. However, in a Brazilian study, *M. abscessus* and *M. chelonae* isolates resumed in infectious keratitis cases, indicating that they are not susceptible to these drugs *in vitro*^(8)^. In a multidrug-resistant case of *M. abscessus* keratitis, topical linezolid was added to the treatment^(9)^. In some cases, flap amputation and keratoplasty may be required^(6)^. Despite all efforts, 50% of the patients with post-LASIK mycobacteria end up with severe visual loss^(2)^.

In conclusion, refractive surgeons should always remember atypical mycobacteria as an etiology of post-LASIK keratitis in patients with interface complications. Unless a subacute atypical mycobacterial infection is early suspected, the diagnosis will be delayed, and pro gressive keratitis and ulceration may cause flap and stromal bed melting. Treatment should therefore be modified accordingly after clinical suspicion.

## References

[1] Randleman JB, Shah RD (2012). LASIK interface complications: etiology, management, and
outcomes. J Refract Surg.

[2] Chang MA, Jain S, Azar DT (2004). Infections following laser in situ ke ra tomileusis: an
integration of the published literature. Surv Ophthalmol.

[3] John T, Velotta E (2005). Nontuberculous (atypical) mycobacterial keratitis after LASIK:
current status and clinical implications. Cornea.

[4] Ko J, Kim SK, Yong DE, Kim TI, Kim EK (2017). Delayed onset Mycobacterium intracellulare keratitis after laser
in situ keratomileusis: A case report and literature review. Medicine (Baltimore).

[5] Nascimento H, Viana-Niero C, Nogueira CL, Martins Bispo PJ, Pinto F, de Paula Pereira Uzam C (2018). Identification of the infection source of an outbreak of
mycobacterium chelonae keratitis after laser in situ
keratomileusis. Cornea.

[6] Moorthy RS, Valluri S, Rao NA (2012). Nontuberculous mycobacterial ocular and adnexal
infections. Surv Ophthalmol.

[7] Daines BS, Vroman DT, Sandoval HP, Steed LL, Solomon KD (2003). Rapid diagnosis and treatment of mycobacterial keratitis after
laser in situ keratomileusis. J Cataract Refract Surg.

[8] Höfling-Lima AL, de Freitas D, Sampaio JL, Leão SC, Contarini P (2005). In vitro activity of fluoroquinolones against Mycobacterium
abscessus and Mycobacterium chelonae causing infectious keratitis after
LASIK in Brazil. Cornea.

[9] Bostan C, Slim E, Choremis J, Boutin T, Brunette I, Mabon M, Talajic JC (2019). Successful management of severe post-LASIK Mycobacterium
abscessus keratitis with topical amikacin and linezolid, flap ablation, and
topical corticosteroids. J Cataract Refract Surg.

